# Effect of Acute Ramadan Fasting on Muscle Function and Buffering System of Male Athletes

**DOI:** 10.3390/healthcare9040397

**Published:** 2021-04-01

**Authors:** Mohamad Fashi, Sajad Ahmadizad, Hadi Nobari, Jorge Pérez-Gómez, Rafael Oliveira, Jorge Carlos-Vivas, Luca Paolo Ardigò

**Affiliations:** 1 Department of Biological Sciences in Sports, Faculty of Sport Sciences and Health, Shahid Beheshti University, Tehran 1983963113, Iran; fashi84.u@gmail.com (M.F.); s_ahmadizad@sbu.ac.ir (S.A.); 2 HEME Research Group, Faculty of Sport Sciences, University of Extremadura, 10003 Cáceres, Spain; jorgepg100@gmail.com (J.P.-G.); jorge.carlosvivas@gmail.com (J.C.-V.); 3 Sports Science School of Rio Maior–Polytechnic Institute of Santarém, 2140-413 Rio Maior, Portugal; rafaeloliveira@esdrm.ipsantarem.pt; 4 Research Centre in Sport Sciences, Health Sciences and Human Development, 5001-801 Vila Real, Portugal; 5 Life Quality Research Centre, 2140-413 Rio Maior, Portugal; 6 Department of Neurosciences, Biomedicine and Movement Sciences, School of Exercise and Sport Science, University of Verona, 37131 Verona, Italy; luca.ardigo@univr.it

**Keywords:** isokinetic, buffering capacity, muscle performance, RPE, HCO_3_^−^

## Abstract

The aim of this study was to investigate the effect of acute Ramadan fasting (RF) on the muscle function and buffering system. Twelve male athletes with 8 years of professional sports experience (age, 23.2 ± 1.3 years, body mass index: 24.2 ± 2.2 kg/m^2^) participated in this study. The subjects were tested twice, 3 weeks after the beginning of RF and 2 weeks after the end RF. Muscle function, buffering capacity, and rating of perceived exertion (RPE) were measured during and after RF by using the Biodex isokinetic machine, blood gas analyzer, and RPE 6–20 Borg scale, respectively. Venous blood samples for pH and bicarbonate (HCO_3_^−^) were measured during and after RF by using the Biodex isokinetic machine, blood gas analyzer, and RPE 6–20 Borg scale, respectively. Venous blood samples for pH and bicarbonate (HCO_3_^−^) were taken immediately after 25 repetitions of isokinetic knee flexion and extension. Measures taken during isokinetic knee extension during RF were significantly lower than those after RF in extension peak torque (t = −4.72, *p* = 0.002), flexion peak torque (t = −3.80, *p* = 0.007), extension total work (t = −3.05, *p* = 0.019), extension average power (t = −4.20, *p* = 0.004), flexion average power (t = −3.37, *p* = 0.012), blood HCO_3_^−^ (t = −2.02, *p* = 0.041), and RPE (Z = −1.69, *p* = 0.048). No influence of RF was found on the blood pH (t = 0.752, *p* = 0.476). RF has adverse effects on muscle function and buffering capacity in athletes. It seems that a low-carbohydrate substrate during RF impairs muscle performance and reduces the buffering capacity of the blood, leading to fatigue in athletes.

## 1. Introduction

Ramadan fasting (RF) is a religious custom in Islam, which requires healthy Muslims to refrain from eating, drinking, smoking, and sexual relations between sunrise and sunset [1]. Generally, adult Muslims observe two main meals, the first meal in the early morning just before sunrise and the second meal at the end of the fasting day after sunset [2]. This eating pattern and length between two meals leads to some changes in sleep and lifestyle rhythms [3].

Several previous studies have showed Ramadan fasting to be associated with significant changes in body weight [4], basic hematologic parameters, blood glucose levels, lipids [4], resting metabolic rate [5], respiratory function [6], and physical activity level [7].

It has been suggested that alterations imposed by the RF may require a reduction in the training load undertaken by Muslim athletes [4,8]. However, many Muslim athletes continue professional sports during Ramadan, even in high-intensity sports. While sports performance depends on the availability of energy (length of each fasting day was approximately 16–17 h) and buffering capacity, there is no precise information for athletes in this regard. Acid–base buffers confer resistance to a change in the pH of a solution when hydrogen ions are added or removed. The most important buffer to maintain acid–base balance in the blood is the bicarbonate buffer, which can affect performance [9]. A significant decrease in the training load allows athletes to delay the fatigue induced by high-intensity training. Some studies have shown that strength and intense aerobic and anaerobic performance are negatively affected by RF [7,8,9,10], whilst others have failed to observe considerable performance decrements following RF [11,12]. For example, Souissi and colleagues (2007) showed that peak power during the Wingate cycling test recorded in the afternoon hours decreased after two weeks of fasting [13]. Similarly, Aziz et al. (2012) have reported a significant reduction in total work during six Wingate tests, which were each followed by 4 min recovery periods [8]. Karli et al. (2007) indicated that RF will not have adverse effects on body composition, anaerobic power or capacity, and lactate metabolism during and after high-intensity exercise in power athletes [12].

RF shifts the substrate balance toward greater use of fatty acids and increases the lipid oxidation of trained athletes both at rest and during exercise [14,15]. In addition, a low-carbohydrate substrate may reduce the buffering capacity of the blood during intense muscular contractions [15], which negatively affects muscle performance. Under these conditions, reduced lactate production is associated with the deactivation of bicarbonate so that the system capacity is reduced [6]. Muscle performance is related to the physical capacity and continued isokinetic muscle contraction [16]. Although, the effects of RF on exercise performance have been investigated extensively [8,13], the effect of acute RF on the muscle function and buffering system of athletes remains unknown. Moreover, most studies have examined the effect of fasting before and after this month or in short periods [8,13,17,18], and so far, no study has examined the effect of fasting after a long period of adaptation and during fasting. For this reason, this study was to evaluate the effect of fasting during RF on the last days when the body has reached a state of adaptation (approximately three weeks) and also after the month of RF, i.e., the time to return to normal, after RF (approximately three weeks). Grantham et al. (2006) reported that there was no difference between isokinetic knee flexor/extensor performance before and after Ramadan [19]. Furthermore, another study examined the effects of fasting on ankle proprioception function by isokinetic test [20], while the present study, in addition to muscle function, for the first time, examined the buffering capacity of athletes during and after fasting. Therefore, the present study was designed to determine whether religious RF has any adverse effects on the muscular function and blood buffering capacity during an all-out isokinetic contraction test.

## 2. Materials and Methods

### 2.1. Participants

Twelve male athletes (age: 23.2 ± 1.3 years, BMI: 24.2 ± 2.2kg/m^2^) voluntarily participated in this study. Inclusion criteria were as follows: (i) participants were non-smokers, (ii) did not have pathological sleep disorders, (iii) did not consume alcohol, (iv) had no lower limb injuries, (v) they had to train regularly at least 8 h/week, and (vi) they had been training for at least 8 years. After familiarization with the protocol, potential risks, and benefits of the study, all participants provided informed written consent prior to the evaluation. The study was conducted and approved by the Shahid Beheshti University Research and Ethics Committee (IR.SBU.REC.1397.055) and the recommendations of Human Ethics in Research were followed according to the Helsinki Declaration.

### 2.2. Study Design

This is a quasi-experimental design that has been done with pre- and post-test. Before starting the first test, height, weight, and BMI were assessed. These variables measurements were performed in the morning [21,22]. The study was carried out in May 2019, where the length of each fasting day was approximately 16–17 h. All subjects performed two exercise tests during (on the 27th day of Ramadan, during-RF) and after Ramadan (14 days after Ramadan in a fed state, after-RF). The length of the fasting period and beyond was similar for the evaluation of athletes. Participants began the warm-up and then performed an isokinetic test to achieve fatigue. Participants were advised to sleep for 8 h and maintain the same pre-determined food intake during the two days before isokinetic test and blood sampling. The amount of nutrients received by participants was calculated using a previously described method [23]. The average temperatures and humidity were around 32 °C and 46%, respectively, for the first trial, and 35 °C and 42%, respectively, for the second trial, which was displayed by a digital thermometer hygrometer.

### 2.3. Isokinetic Test

The exercise trial included knee flexion–extension by using a Biodex System 4 (Biodex Medical System Inc., Shirley, NY, USA). The contraction mode employed was concentric. The dominant leg of each participant was assessed in a single session, at angular velocity of 60°/s; each series contained 25 repetitions [23,24]. The isokinetic variables measured were peak torque, total work, average power, angle of peak torque, and agonist/antagonist ratio. All evaluations were carried out by the same investigator, and the coefficient of variation was below 10%. During the two weeks before and during Ramadan, their exercise rating of perceived exertion (RPE) [25] on the Borg scale (6–20) was recorded. Participants were monitored for their RPE using the grade scale-20 Borg’s scale, which is a valid and reliable scale to estimate the intensity of a session [26,27]. To the question “How intense was your test?” participants answered in the interval of 6 (rest) and 20 (very hard effort). Athletes answered immediately after the end of training during-RF [28,29].

The isokinetic evaluation was preceded by a 2 min warm-up consisting of unloaded knee flexion–extension on the Biodex System. After this, the participants were positioned on the seat, and in order to avoid compensatory movements, they were stabilized with belts around the trunk, pelvis, and the assessed thigh. The hip flexion was set at 85° and the dynamometer axis was aligned with the anatomical axis, related to the lateral femoral epicondyle. The lever arm length was determined as directly above the malleolus height. All calibration procedures and gravity corrections were in accordance with the instruction manual for the equipment. The range of motion was set at 90°, considering a maximum knee extension as 0° and avoiding joint hyperextension. The subjects were instructed not to move their opposite lower limb and to perform maximum effort during all repetitions. Verbal encouragements and visual feedbacks were used to encourage the participants to develop maximum strength.

### 2.4. Blood Samples

Blood samples were obtained immediately after 25 repetitions of isokinetic knee flexion and extension. During this period, participants remained in seated position, and 5 cc of brachial venous blood was obtained, and within 30 min, it was analyzed in a varied blood gas analyzer (Techno Medica, GASTAT 700 series, Yokohama, Japan) to determinate blood pH and bicarbonate (HCO_3_^−^).

### 2.5. Dietary Monitoring

One week before the start of the study, athletes were asked to record and deliver a whole day of total nutrients (carbohydrate, fat, and protein). After calculation with the advice of a nutritionist, participants received oral and written information about the same food intake prior to protocol implementation, and two days before the test, subjects began to record and send in daily food diaries. The daily dietary intake of athletes was measured, and total calorie intake was calculated using Nutrition 4 version 3.5.2 software [30,31]. The caloric information was as follows: total energy (kcal/d) = 2916 ± 150; total protein (g/d) = 109 ± 12; total carbohydrate (g/d) = 401 ± 16; and total fat (g/d) = 97 ± 21.

### 2.6. Statistical Analysis

All data were analyzed by the statistical software Graph Pad PRISM version 6. All data were assessed for normality by using the Shapiro–Wilk test. Between during- and after-RF, the comparisons of isokinetic variables, pH and HCO_3_^−^, were performed by using a paired t-test. The Wilcoxon test was used for the comparison of RPE between during- and after-RF. Cohen’s D effect sizes were determined for each variable and defined as trivial (<0.2), small (≥0.2), moderate (≥0.5), and large (≥0.8). Statistical significance was set at *p* ≤ 0.05. Data are presented as mean ± standard deviation.

## 3. Results

Shapiro–Wilk tests indicated a normal distribution of extension peak torque (*p* = 0.221), flexion peak torque (*p* = 0.426), extension total work (*p* = 0.586), flexion total work (*p* = 0.794), extension average power (*p* = 0.655), flexion average power (*p* = 0.489), angle of extension peak torque (*p* = 0.767), angle of flexion peak torque (*p* = 0.125), agonist/antagonist ratio (*p* = 0.534), HCO_3_^−^ (*p* = 0.433), and pH, (*p* = 0.298).

The changes in peak torque, total work, average power, angle of peak torque, and agonist/antagonist ratio, during-RF and after-RF, are presented in Table 1; and the changes in pH, HCO_3_^−^, and RPE are demonstrated in Table 2. When isokinetic knee measurements were compared, a significant increase was found for extension peak torque (t = −4.72, *p* = 0.002, 18%), flexion peak torque (t = −3.80, *p* = 0.007, 19%), extension total work (t = −3.05, *p* = 0.019, 11%), extension average power (t = −4.20, *p* = 0.004, 17%), and flexion average power (t = −3.37, *p* = 0.012, 30%) after-RF.

In addition, blood HCO_3_^−^ (t = −2.02, *p* = 0.041, 13%) and RPE (Z = −1.69, *p* = 0.048, 9%) were increased significantly after-RF in comparison to during-RF. No significant differences in flexion total work (t = −2.81, *p* = 0.146), angle of extension peak torque (t = −0.936, *p* = 0.380), angle of flexion peak torque (t = 1.18, *p* = 0.274), agonist/antagonist ratio (t = −0.055, *p* = 0.958), and blood pH (t = 0.752, *p* = 0.476) were found between the two measurements.

## 4. Discussion

The purpose of this study was to evaluate the effect of RF on the muscle function and buffering system of male athletes. The main finding of this study was that RF has adverse effects on muscle function and buffering capacity in male athletes.

Although the isokinetic variables including peak torque, total work, and average power are all correlated with the ability of the muscle to contract and generate strength, they evaluate different capabilities of a muscle [32]. The force generated by the quadriceps is evaluated by peak torque, and this ability is associated with the average power. Both parameters explain the critical ability of this muscle group to produce force quickly and efficiently. In contrast, the antagonist activity of the hamstrings is essential for the deceleration of the knee, controlling the joint motion. The performance is indicated by the total work of the flexor muscles, which is associated with torque and changes based on the knee’s range of motion. In general, this variable shows the behavior of the muscle throughout the movement, as opposed to the peak torque [32,33].

Previous studies have reported that RF does not affect maximum short-term performance in active individuals (e.g., explosive power, anaerobic power, and capacity) [11,12,34], and the findings are inconclusive. For example, a study has shown that total work measured during the repeated sprint test did not change significantly during RF [34]. Moreover, the fatigue index recorded during repeated sprint test was not affected by RF [35,36]. On the other hand, a significant increase in absolute and relative peak power values during RF has been reported [12]. It seems that, if strength–power training (i.e., in power athletes) is performed regularly and the total amount of daily food intake, the body water balance, and the daily sleep time were preserved as before RF (meal and sleep pattern), it would not have adverse effects on muscle power [12]. In contrast, others have reported that both aerobic and anaerobic performances are affected by RF [36]. No previous data on the association of the isokinetic variables with muscle function during RF are available. In the present study, we found decreases in extension peak torque (−18%), flexion peak torque (−19%), extension total work (−11%), extension average power (−17%), and flexion average power (−30%) during-RF compared to after Ramadan. It seems possible that these results are due to an absence of food and fluid intake and a decrease of blood glucose, which is positively associated with the total decrease of energy intake during Ramadan, [15,37] and maybe affect isokinetic muscle function variables. In addition, one might anticipate that RF would shift the substrate balance toward a greater use of fatty acids [37], where muscle isokinetic performance depends on carbohydrate as an energy source. Differences in time-course measurement, methods, and duration of calorie restriction can also explain this discrepancy.

Another finding of the current study was the adverse effect of RF on the buffering system during exercise. During high-intensity exercise such as repeated isokinetic contraction, increased blood lactate concentrations require the use of the bicarbonate buffering system to regulate blood pH [38,39]. The low-carbohydrate intake during RF may reduce the buffering capacity of the blood during intense muscular contractions [15]. The present study showed a significant decrease (−13%) in blood HCO_3_^−^ during RF isokinetic performance. However, no significant change was found for blood pH (0%).

Regarding RPE, the findings of the current study do not support previous studies [38,39]. For example, Nugraha et al. (2017) reported that Ramadan intermittent fasting did not affect fatigue when compared to non-fasting control [18]. Additionally, Güvenç (2011) showed that peak RPE scores were similar before and during Ramadan [17]. In the present study, RPE scores during-RF isokinetic performance were higher than after Ramadan (9%), which could also explain a higher perceived exertion of exercise during-RF than after-RF. These results can be associated with the restriction of food intake.

Because of difficulties in recruiting participants during Ramadan, some main limitations of this study were as follows, first, the limited statistical population (sample size), and second, the fact that present study only investigated the acute responses of isokinetic muscle function and buffering system in male athletes. Therefore, future studies should examine a large number of subjects and the long-term effects of repeated isokinetic contraction on the muscle function and buffering system during Ramadan. In addition, there were other limitations that need to be considered. Isokinetic test and blood sampling should be taken before, during, and after RF. As a result, the effects imposed by RF can be better understood with three assessments. Another limitation of the study could be the lack of recording the nutritional information of athletes in the period before the month of fasting, which could provide good comparative information about their daily calorie intake (before, during, and after Ramadan); however, the lack of access to athletes in the period before Ramadan did not allow this. Hence, we strongly recommend that these strategies be considered in future studies.

## 5. Conclusions

In summary, to our knowledge, this is the first study to examine the impact of RF on the isokinetic muscle function and buffering system in male athletes. Based on the findings, we concluded that RF has an adverse effect on the muscle function and buffering system in male athletes. It seems that reducing the carbohydrate substrate of athletes’ muscles during fasting reduces their performance and the buffering capacity of the blood during fasting cross-sectionally, which in turn leads to faster fatigue for the athlete. However, after fasting, the RPE decreased, so it is recommended to coaches and Muslim athletes that they can maintain or improve the mentioned variables by continuing the program of training sessions during Ramadan and after Ramadan as well.

## Figures and Tables

**Table 1:** Mean ± standard deviation values of isokinetic variables at 60°/s during (during-RF) and after Ramadan fasting (after-RF).

Variables	Extension— During-RF	Extension— After-RF	Effect Size	*p*	Flexion—During-RF	Flexion—After-RF	Effect Size	*p*
			**Cohens D**				**Cohens D**	
Peak torque (Nm)	220.7 ± 10.5	259.8 ± 6.3	1.6 L	0.002 *	97.2 ± 4.4	115.7 ± 4.7	1.4 L	0.007 *
Total work (J)	3885.9 ± 96.8	4330.9 ± 100.8	1.6 L	0.019 *	1600.5 ± 146.9	1948.0 ± 105.7	1.0 L	0.146
Average power (W)	114.7 ± 4.7	134.6 ± 4.4	1.6 L	0.004 *	45.9 ± 4.3	59.8 ± 3.2	1.3 L	0.012 *
Angle of peak torque (°)	66.5 ± 1.6	68.6 ± 1.3	0.5 M	0.380	57.5 ± 3.6	52.6 ± 2.0	−0.6 M	0.274
A/AN ratio (%)	44.6 ± 2.7	44. 8 ± 2.0	0.03 T	0.958	-	-	-	-

Abbreviations: A/AN ratio = agonist/antagonist ratio; T = trivial; S = small; M = moderate; L = large. * Indicates a significant difference between the during and after Ramadan fasting tests (*p* < 0.05).

**Table 2:** Mean ± standard deviation values of buffering variables during (during-RF) and after Ramadan fasting (after-RF).

Variables	During-RF	After-RF	Effect Size (Cohens D)	*p*
pH	7.3 ± 0.02	7.2 ± 0.01	−0.4 S	0.476
HCO_3_^−^	28.0 ± 1.1	31.7 ± 2.2	1.3 L	0.041 *
RPE	17.1 ± 0.3	15.6 ± 0.2	−5.5 L	0.048 *

Abbreviations: HCO_3_^−^ = bicarbonate; RPE = rating of perceived exertion; T = trivial; S = small; M = moderate; L = large. * Indicates a significant difference between the during and after Ramadan fasting tests (*p* < 0.05).

## Data Availability

The datasets used and/or analyzed during the current study are available from the corresponding author on reasonable request.
